# Differential associations of ankle and brachial blood pressures with diabetes and cardiovascular diseases: cross-sectional study

**DOI:** 10.1038/s41598-021-88973-3

**Published:** 2021-04-30

**Authors:** Hema Viswambharan, Chew Weng Cheng, Kirti Kain

**Affiliations:** 1 Leeds Institute of Cardiovascular and Metabolic Medicine, Faculty of Medicine and Health, University of Leeds, Leeds, LS2 9JT UK; 2 NHS England & NHS Improvement (North East and Yorkshire), Quarry Hill, Leeds, LS2 7UE UK

**Keywords:** Biomarkers, Health care, Medical research

## Abstract

Increased brachial systolic blood-pressure (BP) predicts diabetes (T2DM) but is not fully effective. Value of absolute ankle systolic BP for T2DM compared to brachial systolic BP is not known. Our objectives were to assess independent relationships of ankle-systolic BP with T2DM and cardiovascular disease in Europeans and south Asians. Cross-sectional studies of anonymised data from registered adults (n = 1087) at inner city deprived primary care practices. Study includes 63.85% ethnic minority. Systolic BP of the left and right-brachial, posterior-tibial and dorsalis-pedis-arteries measured using a Doppler probe. Regression models’ factors were age, sex, ethnicity, body mass index (BMI) and waist height ratio (WHtR). Both brachial and ankle systolic-BP increase with diabetes in Europeans and south Asians. We demonstrated that there was a significant positive independent association of ankle BP with diabetes, regardless of age and sex compared to Brachial. There was stronger negative association of ankle blood pressure with cardiovascular disease, after adjustment for BMI, WHtR and ethnicity. Additionally, we found that ankle BP were significantly associated with cardiovascular disease in south Asians more than the Europeans; right posterior tibial. Ankle systolic BPs are superior to brachial BPs to identify risks of Type 2DM and cardiovascular diseases for enhanced patient care.

## Introduction

Ninety percent of people with prediabetes are unaware of their condition and 30% of patients will have cardiovascular diseases, retinal, renal, neural complication of type 2 diabetes (T2DM) at the time of diagnosis of^1^. Therefore, it is imperative that timely screening of increased risk is carried out with simple, yet efficient tools for prevention of overt T2DM.

Currently, the diabetes screening tools for T2DM are based on age, gender (gestational diabetes), family-history of T2DM (FHoD), high brachial blood-pressure (BP), ethnicity, physical activity and body-mass-index (BMI). These do not perform well and miss 50% of patients with T2DM^2,3^. Ethnicity an underlying risk factor, since the risk profile of south Asians (originally from Indian, Pakistan, Nepal, Sri Lanka or Bangladesh) is different^4,5^. Greater increases in ankle systolic BP and cardiovascular disease have been reported in south Asians compared to Europeans with a history of T2DM^6,7^.

Type 2 diabetes is more closely associated with metabolic or visceral obesity and waist-to-height-ratio (WHtR) and insulin resistance^8^. Cardiovascular risk in south Asians is principally due to greater hyperglycaemia^9,10^. Insulin resistance-related local vascular changes are more common in the lower limb than the upper limb^11^.

We hypothesized that ankle systolic BP will be a more significant discriminator for T2DM and cardiovascular disease than brachial systolic BP, especially in south Asians.

## Results

### Characteristics of participants

The characteristics of the participants were classified as having (1) no T2DM & no cardiovascular disease (2) T2DM (3) cardiovascular disease (4) T2DM & cardiovascular disease (Tables 1 and 2)^12^. Interestingly, we noted that south Asians were younger than Europeans across all four subsets of health conditions. The percentage with known hypertension was greater in Europeans than south-Asians in all four subsets. Similar results were observed for measured raised brachial systolic BP. Furthermore, ankle systolic BP was higher in patients with T2DM (diagnosed at an average age of 48 years in south Asians and 58 years in Europeans) (Tables 1 and 2).

**Table 1:** Descriptive characteristics of UK Europeans with or without diabetes and /or cardiovascular disease.

Europeans Variables	None n = 185	Diabetes n = 56	Cardiovascular disease n = 87	Diabetes + cardiovascular disease n = 65
Age, yrs	48 (16)	65 (12)	65 (14)	68 (14)
Male%	38	54	57	58
Current smoking%	35	34	29	29
Alcohol%	19	29	19	19
Hypertension%	23	80	64	74
Hyperlipidemia%	17	62	46	62
Age when diagnosed diabetes, yrs		58 (12)		60 (13)
Years of diabetes		7 (5)		8 (5)
^†^BMI kg/m^2^	28 (6)	31 (6)	28 (6)	31 (5)
^‡^Waist height ratio	0.56(0.09)	0.60 (0.08)	0.58 (0.07)	0.63 (0.08)
^†^Systolic brachial BP, mmHG	127 (19)	143 (21)	139 (19)	138 (19)
^‡^Systolic ankle BP, mmHg,	148 (28)	158 (35)	144 (37)	157 (31)
Total cholesterol, mg/dl	5.1 (1.1)	4.5 (1.1)	4.5 (1.1)	3.84 (1.0)
Triglycerides, mg/dl	1.90 (1.1)	2.20 (1.2)	1.6 (0.8)	2.27 (1.3)
HDL cholesterol, mg/dl	1.4 (0.4)	1.2 (0.3)	1.4 (0.5)	1.1 (0.3)
BP lowering agents%	20	77	64	81

Values are mean (SD). ^†^standard indicators of concomitant disease. ^‡^ non-standard characteristics.

**Table 2:** Descriptive characteristics of South Asians with or without diabetes and /or cardiovascular diseases.

South Asians Variables	None n = 348	Diabetes n = 153	Cardiovascular disease n = 67	Diabetes + Cardiovascular disease, n = 126
Age, yrs	39 (13)	57 (13)	46 (13)	62 (11)
Male%	34	49	27	48
Smoking%	14	10	12	12
Alcohol%	4	5	3	5
Hypertension%	10	54	24	73
Hyperlipidemia, %	6	53	15	69
Age of diagnoses diabetes, yrs		48 (13)		50 (12)
Years of diabetes		9(7)		12 (7)
^†^BMI kg/m^2^	27 (6)	31 (6)	29 (5)	30 (6)
^‡^Waist height ratio	0.56 ( 0.09)	0.63 (0.08)	0.60 (0.09)	0.63 (0.08)
^†^Systolic brachial BP, mmHG	119 (17)	134 (18)	123 (14)	132 (19)
^‡^Systolic ankle BP, mmHg	133 (26)	152 (24)	138 (29)	149 (35)
Total cholesterol, mg/dl	4.9(0.9)	4.4 (1.1)	4.9(1.2)	4.1 (0.9)
Triglycerides, mg/dl	1.8 (0.9)	2.5 (2.1)	2.1(1.0)	2.3 (1.2)
HDL cholesterol, mg/dl	1.3 (0.6)	1.1(0.3)	1.4 (0.8)	1.2 (0.3)
BP lowering agents %	9	56	28	81

Values are mean (SD). ^†^Standard indicators of concomitant disease. ^‡^Non-standard characteristics.

### Systolic BP were associated with cardiovascular disease and diabetes status in Europeans

The linear regression models using beta unstandardized coefficients (B) were used to estimate the association of six systolic BP levels to cardiovascular disease and T2DM status (Table 3). There were significant linear association between both systolic brachial, as well as the dorsalis pedis BP and cardiovascular disease (brachial right, P < 0.001; brachial left, P < 0.001; dorsalis pedis right leg, P < 0.018; dorsalis pedis left leg, P < 0.001). Among these four BP measurements, dorsalis pedis left leg was highly associated with cardiovascular disease (*B* = 7.82 [3.82–11.81]). Intriguingly, all six systolic BP (brachial, dorsalis pedis and posterior tibial) were significantly associated with T2DM. All beta coefficients were positive, indicating a positive association between BP and T2DM. In General model B, posterior tibial right leg and dorsalis pedis left leg are both strongly associated with T2DM (*B* = 14.63 [10.67–18.58] and *B* = 14.30 [10.49–18.12]). Therefore, ankle systolic and brachial systolic BP were strongly correlated with cardiovascular disease and T2DM in Europeans.

**Table 3:** Beta (*B*) coefficients for six systolic blood pressure levels to cardiovascular disease and diabetes status using linear regression model.

Systolic blood pressure levels	General model A				General Model B				
	*n*	*B *(95% CI)	*P*-value	R^2^	*n*		*B *(95% CI)	*P*-value	R^2^
Brachial right	928	6.26 (3.34–9.17)	** < 0.001**	0.019	926		10.49 (7.70–13.28)	** < 0.001**	0.056
Brachial left	721	6.10 (3.17–9.03)	** < 0.001**	0.023	719		10.82 (8.03–13.61)	** < 0.001**	0.075
Posterior tibial right leg	1082	3.54 (− 0.62–7.70)	0.095	0.003	1080		14.63 (10.67–18.58)	** < 0.001**	0.047
Posterior tibial left leg	1074	3.82 (− 0.09–7.74)	0.056	0.003	1072		11.49 (7.74–15.25)	** < 0.001**	0.033
Dorsalis pedis right leg	1071	4.84 (0.84–8.84)	**0.018**	0.005	1069		13.10 (9.28–16.92)	** < 0.001**	0.041
Dorsalis pedis left leg	1069	7.82 (3.83–11.81)	** < 0.001**	0.014	1067		14.30 (10.49–18.12)	** < 0.001**	0.048

General model A: cardiovascular disease status; General model B: Diabetes status.

### Negative association between blood pressures and cardiovascular disease status

To further extend the analysis from general model presented in Table 3, the linear regression models were divided to three models adjusted to the respective parameters; model 1, adjusted for age and sex; model 2, adjusted to age, sex, BMI, WHtR and six ethnicity groups; and model 3, adjusted to age, sex, BMI, WHtR and two ethnic groups as categorical variable (Table 4). In model 1, after adjusting for age and sex, no association was found between right brachial, left brachial, right dorsalis pedis and left dorsalis pedis (P > 0.05) BP. However, there was a negative association of right posterior tibial BP with cardiovascular disease (*B* = − 4.98 [− 9.22 to − 0.075]). In model 2 and model 3, the analyses were similar to model 1 but additionally adjusted to BMI, WHtR and ethnicity. Consistently, we found that the posterior tibial right leg BP negatively associated with cardiovascular disease in model 2 and model 3 (P < 0.05). Additionally, when the ethnicity was restricted to European and south Asian populations, brachial right, left posterior tibial and left dorsalis pedis BP were negatively associated with cardiovascular diseases. Right posterior tibial BP was significantly different across all 3 models. The association of ankle BP was more significant than brachial BP with cardiovascular diseases. Therefore, ankle BP showed independent negative association with CVD.

**Table 4:** Associations between blood pressures and cardiovascular disease status.

Systolic blood pressure levels	Model 1			Model 2			Model 3		
	*n*	*B *(95% CI)	*P*-value	*n*	*B* (95% CI)	*P*-value	*n*	*B* (95% CI)	*P*-value
Brachial right	928	− 1.85 (− 4.59–0.89)	0.186	925	− 2.50 (− 5.23–0.22)	0.072	918	− 2.84 (− 5.56 to − 0.11)	**0.042**
Brachial left	721	0.17 (− 2.54–2.88)	0.900	719	− 0.42 (− 3.14–2.29)	0.759	712	− 0.80 (− 3.52–1.92)	0.565
Posterior tibial right leg	1082	− 4.98 (− 9.22 to − 0.075)	**0.021**	914	− 5.10 (− 9.68 to − 0.53)	**0.029**	906	− 7.12 (− 11.62 to − 2.61)	**0.002**
Posterior tibial left leg	1074	− 3.70 (− 7.70–0.29)	0.069	904	− 4.20 (− 8.54 to − 0.15)	0.058	896	− 6.11 (− 10.40 to − 1.83)	**0.005**
Dorsalis pedis right leg	1071	− 3.42 (− 7.48–0.64)	0.099	916	− 3.84 (− 8.22–0.55)	0.086	909	− 5.17 (− 9.54 to − 0.80)	**0.020**
Dorsalis pedis left leg	1069	− 0.80 (− 4.81–3.21)	0.696	914	− 1.64 (− 5.92–2.64)	0.452	907	− 2.77 (− 7.03–1.48)	0.201

Adjusted model: Ankle blood pressure is negatively associated with cardiovascular disease status. Model 1: adjusted to age and sex; Model 2: adjusted age, sex, BMI, waist height ratio, ethnicity; Model 3: adjusted age, sex, BMI, waist height ratio and European and South Asian groups.

### Positive associations between ankle blood pressures and diabetes status

Based on the General Model B in Table 3, the analysis was adjusted for age and sex (model 1) and BMI, WHtR and ethnicity (model 2) to analyse the association of ankle BP with T2DM. (Table 5). Additionally, ethnic group was subcategorised into Europeans and south Asians (model 3). In model 1, positive associations were evidenced in right posterior tibial (*B* = 6.06 [1.73–10.40]) and left dorsalis pedis BP (*B* = 4.26 [0.14–8.37]). However, no association was found when the analyses were adjusted for BMI, WHtR and ethnicity. Therefore, there was a significant positive independent association of ankle BP with T2DM, regardless of ethnicity, BMI and WHtR, indicating that ankle BP is better discriminator than brachial for T2DM.

**Table 5:** Associations between blood pressures and diabetes status: Ankle pressure is a better discriminator than brachial for diabetes.

Systolic blood pressure levels	Model 1			Model 2			Model 3		
	*n*	*B* (95% CI)	*P*-value	*n*	*B* (95% CI)	*P*-value	*n*	*B* (95% CI)	*P*-value
Brachial right	926	0.37 (− 2.47–3.22)	0.796	923	0.10 (− 2.87–3.07)	0.949	916	0.25 (− 2.73–3.23)	0.871
Brachial left	719	1.63 (− 1.30–4.57)	0.275	717	1.31 (− 1.78–4.39)	0.406	710	1.49 (− 1.60–4.59)	0.343
Posterior tibial right leg	1080	6.06 (1.73–10.40)	**0.006**	911	4.35 (− 0.55–9.24)	0.082	904	4.45 (− 0.46– 9.37)	0.076
Posterior tibial left leg	1072	3.11 (− 1.01–7.23)	0.138	901	2.73 (− 1.95–7.41)	0.253	894	2.87 (− 1.83–7.57)	0.232
Dorsalis pedis right leg	1069	4.15 (− 0.01–8.30)	0.050	913	3.74 (− 1.00–8.49)	0.122	907	4.00 (− 0.77–8.77)	0.100
Dorsalis pedis left leg	1067	4.26 (0.14–8.37)	**0.043**	911	3.84 (− 0.80–8.47)	0.104	905	4.00 (− 0.65–8.65)	0.092

Model 1: adjusted to age and sex; Model 2: adjusted age, sex, BMI, waist height ratio, ethnicity; Model 3: adjusted age, sex, BMI, waist height ratio and European and South Asian groups.

### Associations between blood pressures and cardiovascular disease status in European and South Asian populations

Based on model 3 in Table 4 we, further refined the results by investigating the associations between BP and cardiovascular disease; specifically, in Europeans and south Asians (Table 6). Generally, no association was found in all analyses involving Europeans. Ankle BP were significantly associated with cardiovascular disease in south Asians; right posterior tibial (*B* = − 7.05 [− 12.26 to − 1.83]), left posterior tibial (*B* = − 5.05 [− 10.00 to − 0.11]) and right dorsalis pedis (*B* = − 5.53 [− 10.58 to − 0.47]) BP. This result indicate that ankle BP is a better determinant than brachial BP for T2DM and CVD in this cross-sectional study.

**Table 6:** Associations between blood pressures and cardiovascular disease status in European and South Asian populations: Ankle pressure is a better determinant than brachial pressure for cardiovascular disease, after adjusted for ethnicity.

Systolic blood pressure levels	Ethnicity	Linear regression model			
		*n*	*B* (95% CI)	*P*-value	R^2^
Brachial right	European	333	− 2.48 (− 7.36–2.40)	0.318	0.236
	South Asian	585	− 3.27 (− 6.55 to − 0.00)	0.050	0.256
Brachial left	European	252	− 0.85 (− 5.88–4.18)	0.740	0.231
	South Asian	460	− 0.90 (− 4.12–2.32)	0.584	0.258
Posterior tibial right leg	European	329	− 6.94 (− 15.36–1.49)	0.106	0.116
	South Asian	577	− 7.05 (− 12.26 to − 1.83)	**0.008**	0.183
Posterior tibial left leg	European	318	− 7.91 (− 16.09–0.27)	0.058	0.105
	South Asian	578	− 5.05 (− 10.00 to − 0.11)	**0.045**	0.181
Dorsalis pedis right leg	European	328	− 4.26 (− 12.49–3.97)	0.309	0.079
	South Asian	581	− 5.53 (− 10.58 to − 0.47)	**0.032**	0.164
Dorsalis pedis left leg	European	324	− 5.04 (− 12.80–2.72)	0.202	0.120
	South Asian	583	− 1.41 (− 6.48–3.66)	0.585	0.161

Blood pressure levels adjusted to age, sex, BMI and waist height ratio.

## Discussion

Our datasets provide a novel insight that ankle systolic BP is a statistically significant, independent determinant for T2DM, especially in south Asians when compared to brachial. Ankle systolic BP are also associated with cardiovascular disease more than the brachial.

This is a first study of comparison of associations of brachial and ankle BP with diabetes and cardiovascular disease. We demonstrated a significant and positive independent association of ankle BP with diabetes, regardless of ethnicity. Our findings are biologically plausible since metabolic alterations due to insulin resistance cause structural and functional changes in arteriolar and capillary systems and are more pronounced in the lower extremities^13–16^. Peripheral arterial resistance is possibly increased in the arterioles of the lower limb, which may lead to the increase in BP in the arteries of the legs prior to the onset of prediabetes^15^. Furthermore, athero-thrombotic occlusive changes in arteries leads to lower leg amputations and adverse pathological changes rarely affect upper limbs^14^. Increased brachial BP are probably reflective of only central pathological changes, whereas increased ankle BP might be indicative of initial local lower limb vascular perturbations^15^. These local changes may even precede changes in glycosylated haemoglobin^8^.

In the current study, we observed a negative association of ankle BP with cardiovascular disease, independent of age, gender, BMI, WHtR and ethnicity^17^.

Ankle BP were significantly associated with cardiovascular disease status in south Asian population compared to Europeans^6^. It is plausible that ankle systolic BP are highly related to insulin resistance than brachial BP in south Asians. In south Asians, insulin resistance has been observed to be mainly responsible for myocardial infarction and stroke^16,18,19^. It is well-documented that south Asians probably have predominately, micro-circulatory adverse perturbances compared to macro-circulatory changes, as evidenced by very low prevalence of both peripheral arterial disease (defined by ankle brachial index of < 0.9) and abdominal aortic aneurysms^20,21^. It is plausible that although the prevalence of hypertension is not higher in south-Asians without diabetes, the BP increases (higher in the ankle than in the brachial arteries) are related to increased risks of cardiovascular diseases^22^. South Asians have increased diabetic nephropathy, which might also contribute to the development of differential increases in blood pressures of arms and legs^19^. The United Kingdom government funds general practitioners to screen subjects for cardiovascular disease, over the age of 40 within the National Health Service Health Check programme^23^. However, screening at this age is too late for south Asians since their risks for cardiovascular diseases, are already established.

Technologically advanced equipment is also available currently for quick and easy automatic systolic BP measurement in lower limbs^24^. A threshold for ankle systolic BP that predicts a high risk of developing diabetes (or represents HbA1c in the risk of prediabetes range), and assessment of the strength of the associations between WHtR and ankle systolic BP will be clinically useful especially in young south Asians^25^. Blood pressure measurement is non-invasive and more practical as a population screening tool even in low and middle income countries as it can be done easily using a Doppler machine.

This was the first operational and observational study in primary care to investigate if ankle systolic BPs were better independent discriminators of T2DM and cardiovascular disease compared to brachial. Outcome measures were complete in this cohort, to allow for comprehensive analysis. We compared our results with those of relatively small number of Europeans to learn the impact of south Asian ethnicity increasing the chances of greater impact.

We did not have diet details which might have strengthened our risk models. We cannot establish any causal relationships in the current cross-sectional study design. The left–right bias in blood pressures and differences in proximal and distal arterial blood pressures was not adjusted for in our analysis.

### Implications for research and/or practice

Therefore, ankle systolic BPs are superior to brachial BPs to identify risks of Type 2DM and cardiovascular diseases for enhanced patient care. It is important that the novel utilisation of ankle BP in scoring systems for early, cost-effective detection of at-risk individuals in the general population is tested further; especially since early detection is imperative for Covid-19 patients, as well^26^.

## Methods

### Participants and study design

The design was cross-sectional and conducted as described before^6^. The project was performed in accordance with the Strengthening the Reporting of Observational Studies in Epidemiology statement^12^.

### Patient and public involvement

Although patients were not directly involved in the development of the research question, design and outcome measures of this study, patients who participated have helped in the recruitment process of the study by informing their family and relatives. The results will be disseminated to study participants by local Clinical Commissioning Group reports.

### Clinical assessment

A standard questionnaire was administered to all participants. Cardiovascular disease was defined as previous history of any of the following: myocardial infarction, stroke, transient ischaemic attack, peripheral arterial disease, angioplasty, coronary artery bypass surgery or heart failure. Participants with a diagnosis of diabetes were identified by review of medical records. History of T2DM was established according to WHO criteria 1999. T2DM duration, cardiovascular risk factors and complications were recorded. Hypertension and hyperlipidaemia were defined as either previously diagnosed or currently taking antihypertensive or cholesterol-lowering medications. Height and weight were recorded (to the nearest 0.01 m and kg, respectively) to calculate each participant’s BMI, calculated as weight/(height)^2^ (kg/m^2^). Waist circumference was measured at the midpoint between the lowest rib and iliac crest (to nearest cm) to calculate each participant’s WHtR.

### Blood pressure measurements

Participants were rested in the supine position for 5 min before BP measurements were taken using appropriately sized cuffs and a handheld continuous wave Doppler instrument (Huntleigh Super Dopplex II, Huntleigh Healthcare, Cardiff, UK) with an 8 MHz probe and a calibrated mercury sphygmomanometer (http://www.framinghamheartstudy.org/share/protocols/ankle1_8s_protocol.pdf).

Brachial systolic BP was taken in both arms by placement of the cuff in the upper arm and measuring the systolic BP by placing the Doppler probe over the brachial artery in the antecubital fossa. For ankle systolic BP, the blood pressure cuff was positioned superior to the medial malleolus in each leg. Systolic BP was measured over the dorsalis pedis and posterior tibialis arteries on right and left limbs. For each BP measurement, the cuff was inflated until the pulse was no longer audible. The cuff was inflated a further 20 mmHg above the approximate value, at which the pulse was obliterated then deflated slowly, with the pressure being recorded when the pulse became audible using the Doppler probe again.

Should the strength of the relationship between disease (T2DM or cardiovascular disease) depend on factors, such as ethnicity or gender our conclusions will depend on the variations among the participants in our project. We have deliberately recruited from a population enriched for south Asians and those with the relevant conditions studied. Consequently, the effects in our sample may be stronger than those in the general population.

### Statistical analysis

For descriptive purposes, patient characteristics based on disease status and ethnicity were summarised and tabulated. Continuous measurements were presented as means ± SDs, categorical measures as absolute numbers and percentages. Data with p values less than 0.05 was considered significantly different and exact values, presented.

R Software version 4.0.2 was used to perform all analysis. Descriptive demographic characteristics were calculated for all subjects. In the first analysis, linear regression models were built to assess the association between six systolic BP and cardiovascular disease and T2DM status. These univariate analyses were named “General model”. The systolic BP measurements were continuous variable while the cardiovascular disease and T2DM status was categorical variable. To extend the analysis, the general model analyses were adjusted to a set of covariates such as age, sex, BMI, waist height ratio and ethnicity. Model 1 was adjusted for age and sex. Model 2 linear regression analysis was adjusted to age, sex, BMI, WHtR and six ethnicity groups. Model 3 was adjusted to age, sex, BMI, WHtR and two major ethnicity groups (European and South Asian). In the final analysis, we aimed to investigate the association of between six systolic BP and cardiovascular disease and T2DM status in two ethnic groups (European and South Asian). A two-tailed *P* < 0.05 threshold was set as the significant level for all analysis. Since missing data was low, complete case analysis without imputations was carried out.

### Ethical approval

The project was approved by the Leeds-Bradford Research Ethics Committee (REC 10/H1302/28) and local Research and Development. All methods and experimental protocols were carried out in accordance with the Declaration of Helsinki (2013). In addition, all methods and experimental protocols were reviewed and approved by the Leeds-Bradford Research Ethics Committee, equivalent to the present 2016 Integrated Research Application System UK. Written informed consent was obtained from each participant according to Good Clinical Practice guidelines. The response rate for recruitment was 60%.

A purposive sample of 1087 consecutive consenting patients were recruited at an inner-city primary care practice in West Yorkshire, UK, as described previously^6^. Indians, Pakistanis and Bangladeshis, White Europeans, other Asians and different ethnics (e.g. Afro-Caribbean), were the 6 groups. Recruitment of adults was consecutive from all primary care clinics. Inclusion criteria were participants aged ≥ 18 years. There were 694 south Asians (originally from India, Pakistan, Bangladesh or one or more of their grandparents born in one of these countries).

Participant’s ethnicity was based on electronic medical record data or ascertained from demographic data collected at recruitment which were self-reported, surname assignment and country of birth of grandparents. All clinical assessments (i.e., medical history and measurements) were performed at the same visit.

## Data Availability

The datasets generated during and/or analysed during the current study are not publicly available since patient permission was not sought for the sharing of data, at the time of recruitment.
